# Profile of Tabriz Older People Health Survey (TOPS-2019): a representative community-based cross-sectional study

**DOI:** 10.1038/s41598-022-22710-2

**Published:** 2022-10-25

**Authors:** Mostafa Araj-Khodaei, Sarvin Sanaie, Seyed Aria Nejadghaderi, Mark J. M. Sullman, Sirous Samei-Sis, Somaiyeh Taheri-Targhi, Zahra Yousefi, Hossein Matlabi, Saeid Safiri, Akbar Azizi-Zeinalhajlou

**Affiliations:** 1 Research Center for Integrative Medicine in Aging, Aging Research Institute, Tabriz University of Medical Sciences, Tabriz, Iran; 2 School of Medicine, Shahid Beheshti University of Medical Sciences, Tehran, Iran; 3 Systematic Review and Meta‐Analysis Expert Group (SRMEG), Universal Scientific Education and Research Network (USERN), Tehran, Iran; 4 Department of Social Sciences, University of Nicosia, Nicosia, Cyprus; 5 Department of Life and Health Sciences, University of Nicosia, Nicosia, Cyprus; 6 Student Research Committee, Aging Research Institute, Tabriz University of Medical Sciences, Tabriz, Iran; 7 Research Center of Psychiatry and Behavioral Sciences, Aging Research Institute, Tabriz University of Medical Sciences, Tabriz, Iran; 8 Department of Psychology, Faculty of Educational Sciences and Psychology, University of Tabriz, Tabriz, Iran; 9 Department of Geriatric Health, Faculty of Health Sciences, Tabriz University of Medical Sciences, Tabriz, Iran; 10 Neurosciences Research Center, Aging Research Institute, Tabriz University of Medical Sciences, Tabriz, Iran; 11 Department of Community Medicine, Faculty of Medicine, Tabriz University of Medical Sciences, Tabriz, Iran; 12 Physical Medicine and Rehabilitation Research Center, Aging Research Institute, Tabriz University of Medical Sciences, Tabriz, Iran

**Keywords:** Psychology, Medical research, Risk factors

## Abstract

Population aging and its consequences are a substantial global concern. The growth in the number of older people is one of the most important factors increasing the burden of non-communicable diseases (NCDs) on society. The Tabriz Older People Health Survey aimed to understand the socio-demographics, health-related behaviors, and health profile of older adults. This cross-sectional study was conducted on a representative sample of 1362 community-dwelling older adults in Tabriz, the most populated city in northwest Iran. The study used probability proportionate to size sampling and the data collection was undertaken in each participants’ place of residence from July 2019 to January 2020. Trained interviewers administered the questionnaire, which measured each participant’s socio-demographics, health-related behaviors, and health profile. The sample of 1362 participants consisted of 56.4% women and 54.4% were young older people (60–69 years old). Almost half of the sample were completely illiterate. There was no daily walking in 13.3% of the sample, with women reporting a more sedentary lifestyle than men. Almost 10% of the participants (n = 135, 9.9%) were current smokers, which was higher among men (20.9% vs. 1.5%) and women made up over 88% of those living alone. In terms of sleep quality score (men: 4.63 ± 2.70, women: 5.97 ± 2.93), anxiety (men: 5.79 ± 4.70, women: 7.59 ± 5.51), depression (men: 9.54 ± 3.20, women: 10.63 ± 3.09), and social support (men: 23.65 ± 4.50, women: 22.69 ± 4.77), men were significantly better than women. There were also significant sex differences between women and men in the prevalence of diabetes (31.6% vs. 19.5%) and hypertension (86.5% vs. 73.4%). Furthermore, overall hypertension was the most common underlying disease (81.0%). Older women were significantly worse off than older men, in terms of social and disability-related, as well as having a higher burden from several NCDs. The results of this study might help regional health policymakers to identify targets for improving the health status among community-housed geriatrics.

## Introduction

The world is currently facing an unprecedented situation, which is the rapid growth in the number and proportion of older adults in the population^1^. Although developed countries have already begun to face this phenomenon, the United Nations (UN) projects that the most rapid aging will occur in developing countries. Iran is one of the countries that will experience rapid population aging, with those aged over 60 years old expected to increase from 10% of the population in 2016 to more than 33% by 2050^2^.

Population aging has already had a profound impact on society, including contributing to rising healthcare costs^3,4^. The increasing burden of non-communicable diseases (NCDs) is a major global public health challenge, with older adults having the highest prevalence of most NCDs and more often require treatment^5^. According to the Global Burden of Disease (GBD) study, in 2019 78.1% of all disability-adjusted life-years (DALYs) in Iran were caused by NCDs, compared with 43.0% in 1990^6^. The prevalence of NCDs also differ according to sociodemographic factors, such as educational level, income, age, and sex. Although the rates of NCDs are increasing globally, due to population aging^7^, the largest increases are being found for cardiovascular diseases^8^, cancers^9^, psychiatric disorders^10^, and musculoskeletal disorders^11^. Furthermore, substantial sex differences were found, which may be due to different levels of exposure to important risk factors^12^. These age-and sex-differences in the burden of NCDs have also been observed in the Middle East region. For instance, studies measuring the burden of mental disorders in the Eastern Mediterranean Region found that mental disorders were much more common in older adults than in the middle-aged and younger population^13^, and that the sex differences in the burden of mental disorders were more pronounced in this region than at the global level^11^.

It is important to note that the burden of NCDs in the above-mentioned studies have all been estimated separately, although most older people suffer from more than one disease. Multi-morbidity has largely been neglected in the studies reporting the burden of diseases in this population, hence the burden of NCDs may be underestimated in these general studies. Multi-morbidity is a substantial challenge for health systems worldwide^14^. The high prevalence of multi-morbidity among older people may have substantial financial implications over the coming decades, especially in low- and middle- income countries^15^. Multi-morbidity is mostly driven by age, with the proportion of the population who suffer from multi-morbidities increasing progressively with population aging^16^. The proportion of people aged 65 years and above in the United States of America with one or more chronic disease increased from 86.9% in 1998 to 92.2% in 2008^17^. While 54% of people aged > 65 years in England exhibited multi-morbidities in 2015, it is projected to be 68% by 2035^18^. Low physical activity, overweight and obesity, hypertension, and dyslipidemia are among the most important risk factors^19^. All of these risk factors are modifiable, and the elimination of these would prevent a substantial proportion of these NCDs^20^. The prevalence of obesity in the population over 18 years of age in Iran increased substantially between 2000 and 2016, reaching about 23% of the population^21^. Furthermore, the prevalence of obesity in Iranian adults aged 50 and above was 21.4%^22^.

In terms of the proportion of older adults in the population, East Azerbaijan has the fifth largest proportion in Iran. Tabriz, as the capital of the province, is the most populous city in northwestern Iran^23^. Tabriz has experienced rapid social and lifestyle changes in recent decades. Along with these changes, the prevalence of known risk factors for NCDs have also increased rapidly. The increasing burden of NCDs is attributable to both an increase in the prevalence of risk factors and the growing number of older people^24^. In 2015, 2.5% of those aged ≥ 60 suffered from malnutrition and 26.7% were at risk of malnutrition in Tabriz^25^. A high prevalence of hypertension has also been reported in a representative sample of community-dwelling older adults in Tabriz, with about 22% being untreated and only 46% were adequately controlled^26^.

NCDs generally have strong social determinants and have shared lifestyle risk factors, many of which are modifiable and amenable to intervention^24^. In addition, the proportion of older adults is growing and so focusing on their situation is increasingly important. Social planning and managing these huge demographic changes, along with their extensive consequences, are vital. However, proper planning and dealing with this phenomenon would not be possible without a comprehensive evaluation of the different aspects of aging. As a result, the Tabriz Older People Health Survey (TOPS) was conducted by the Aging Research Institute (ARI) at the Tabriz University of Medical Sciences (TUOMS). The present study aimed to measure the socio-demographics, health-related behaviors, and the health profile of older people living in Tabriz. These were all reported by sex, since sex differences are large in Eastern Mediterranean Region. The present study will provide information useful for policy makers and health providers, and help to identify ways of improving the health status of these older residents.

## Materials and methods

### Study setting and population

The study was conducted in Tabriz, which is divided into 10 municipal districts. The population included all community-dwelling adults aged ≥ 60 in Tabriz, which numbered 174,158 inhabitants, according to Iran’s most recent Population and Housing Census in 2016^12^. Inclusion criteria were being at least 60 years old and consenting to participate in the study.

### Sample size and sampling method

This was a cross-sectional study of a representative sample of community-dwelling older adults living in Tabriz. The minimum required sample size needed to estimate the prevalence of type II diabetes, depression and hypertension were calculated using data from previous studies^26–28^. At all steps, type I and type II errors were considered to be 0.05 and 0.2, respectively. The initial sample sizes for type II diabetes (n = 1033), depression (n = 717) and hypertension (n = 1044) were different, but the highest (n = 1044) was selected. However, the literature suggests that about 10 cases are needed per variable included, when using multivariable model. Therefore, a final sample size of 1400 was selected, to allow the use of multivariable analyses.

**Figure 1:**
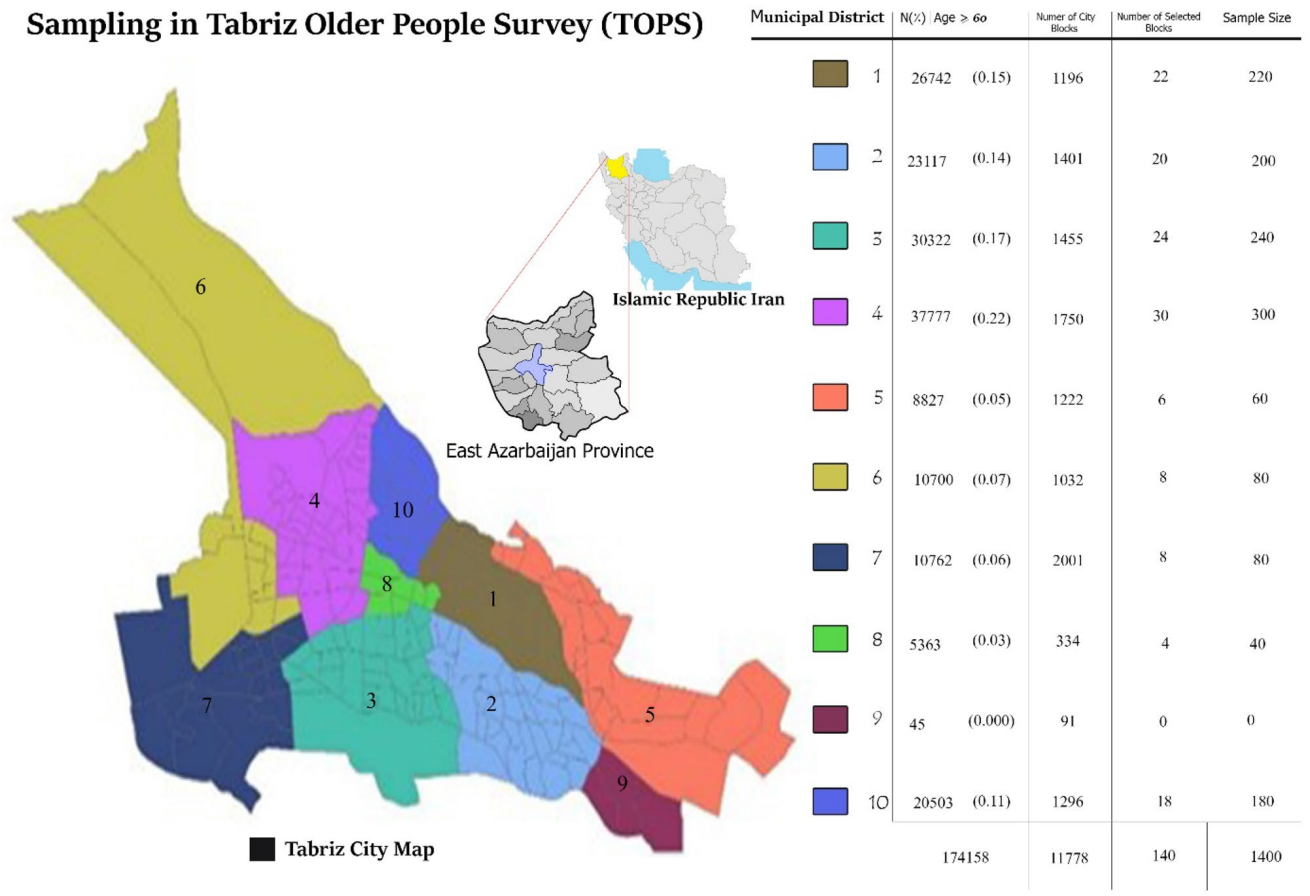
The number and proportion of older adults living in the municipal districts, the allocated sample size, and the number of blocks selected from each district of the ten municipal districts of Tabriz for the Tabriz Older People Health Survey (TOPS) study. (This figure was generated by Adobe Photoshop CC 14.0 and the maps were obtained from Wikipedia.). https://en.wikipedia.org/wiki/Provinces_of_Iran. https://en.wikipedia.org/wiki/East_Azerbaijan_province. https://en.wikipedia.org/wiki/Tabriz_County#/media/File:Tabriz_county.PNG. https://fa.wikipedia.org/wiki/%D9%85%D9%86%D8%A7%D8%B7%D9%82_%D8%B4%D9%87%D8%B1%D8%AF%D8%A7%D8%B1%DB%8C_%D8%AA%D8%A8%D8%B1%DB%8C%D8%B2.

The municipal districts were initially considered as strata. The sampling was conducted using the probability proportionate to size (PPS) sampling method for each municipality district of Tabriz city. PPS consists of two steps which broadly involves selecting clusters and then selecting people from within those clusters. In the first step, larger clusters have a greater chance of being selected, while in the second step individuals in smaller clusters have a higher chance of being selected. However, it is generally thought that the second stage makes up for the first stage, so that each individual in the population has the same probability of being sampled^29^. The information from the latest national census, which divided Tabriz into 10 municipal districts (strata) and 11,778 urban blocks, was used in the sample selection. In the first step the sample size for each strata were calculated and the population data were separated into strata. In order to obtain 10 participants from each block, the number of selected blocks from each strata was determined (Fig. 1). The following process was undertaken to select which city blocks, which were the primary sampling units, to sample from. The older population sizes were listed in such a way that each cluster (city block) had its own older population size listed. Then the cumulative sum of the older population sizes was calculated to get the Sampling Interval (SI) Divide, the total older population of strata was divided by the number of clusters to be sampled. A random number generator (RANDOM.ORG) was then used to generate a number between 1 and the SI to randomly select the first element (Random Start [RS]) in the systematic sampling procedure, in order to avoid sampling error. The first cluster to be sampled contains this cumulative population and other blocks were then systematic randomly selected (Calculating the following series: RS; RS + SI; RS + 2SI; ….; RS + nSI). Finally, 10 older adults were randomly selected and invited to take part in this study from each selected city block (1400 people from 140 city blocks). From the 1400 people selected for the study, 1362 people fully completed the study process.

### Data collection

The data for the TOPS-2019 study were collected by trained interviewers. All interviewers were trained by experts in how to ask questions and complete the questionnaire. The interviews were conducted in the participant’s home and lasted for 60–90 min. A panel of experts, including specialists in gerontology, geriatrics, psychology, sociology, nutrition, Iranian traditional medicine, and allied fields suggested and approved the questions. Face validity was ensured by the panel and the questionnaire was piloted on 50 participants. A number of anthropometric and blood pressure measures were also recorded in the three sessions before and during the interviews. The data collection took place from July 2019 to January 2020, and 1362 individuals aged 60 and above were interviewed, meaning a response rate of 97%. Informed consent was obtained from all participants and they were assured about the confidentiality of all information collected. This study was undertaken in accordance with national regulations and ethical guidelines.

### Procedure and measurements

The questionnaire consisted of several sections and the data for these sections were either measured directly (i.e., anthropometric data and medication) or self-reported during the interview. The first section asked questions about socio-demographic details, including: date of birth, sex, marital status, level of education, family size, health insurance status, residential and living conditions. Income, profession and professional status, which are indicators of socio-economic status, were also asked at the end of the interview in order not to affect the interview process. The remaining sections of the questionnaire consisted of validated scales and tools, which are described below:

1. **Abbreviated mental test score (AMTS)** It is a scale which tests for dementia in older adults, and is currently also used to assess mental confusion and other cognitive impairments. The AMTS has ten questions with one point allocated for each. Scores lower than 8 indicate the presence of cognitive impairment. The Persian version of the AMTS has been found to be valid for examining the cognitive state of older adults^30^.
2. **Socio-economic Status Questionnaire for Urban Households (SES Iran):** This scale was developed in Iran and has been found to have good validity and reliability. The scale is a valuable addition to most health surveys or clinical studies, but can also be used as the main measure in research about health equity and economics ^31^.
3. **Satisfaction with Life Scale (SWLS):** This scale was developed in 1958 by Diener et al. and has been found to have good reliability and validity in many intercultural studies. The scale contains five items, which are answered using a Likert scale, with higher scores indicating greater satisfaction with life^32^. The validity and reliability of the Persian version of the SWLS has been previously demonstrated^33^.
4. **Activities of Daily Living (ADLs):** This scale measures the basic tasks that must be accomplished every day for an individual to thrive. The Katz ADL scale is a widely used instrument that assesses six primary functions, which are: bathing, dressing, going to the toilet, moving around, feeding, and continence^34^. The total score ranges from 0 (very dependent) to 6 (independent). The Persian version of the ADL has been found to be an appropriate and valid measure for assessing the daily activities of older Iranian adults^35^.
5. **Instrumental Activities of Daily living (IADL):** The Lawton IADL scale is the most widely used instrument to measure disability levels and assess functioning in community-dwelling older adults^36^. The IADL measures more complex activities than the ADL and includes: 1- Ability to use a telephone, 2- Shopping, 3- Food preparation, 4- Housekeeping, 5- Laundry, 6- Transportation method, 7- Medication use, and 8- Handling finances. The IADL is answered on a three point Likert scale (1 = unable, 2 = needs assistance, 3 = independent) and the total score ranges from 8 (lowest ability) to 24 (highest ability). The validity of the Persian version of the IADL has previously been confirmed^35^.
6. **Mini Nutritional Assessment-Short Form (MNA-SF):** The MNA-SF is one of the most commonly used screening tools for identifying the risk of malnutrition in older adults. The nutritional status of each older adult is placed into one of three categories, based on the total score, which are: normal nutritional status, risk of malnutrition, and malnourished^37^. Previous research in Iran has shown the MNA-SF to have a high degree of agreement with the full MNA and that it is an effective screening instrument for the rapid detection of nutritional status in community-dwelling older adults^38^.
7. **Anthropometric instruments and conditions:** A Seca portable digital scale (accurate to 100 g), a Seca inelastic measuring tape and a stadiometer (accurate to 0.5 cm) were used to measure each participants’ weight (kg), waist circumference (WC) (cm), hip circumference (HC) (cm), and height (cm). The participants were weighed while wearing light clothes and without shoes. Using the measuring tape, the WC measurement was taken at the WHO recommended point (i.e. the midpoint between the last rib and the iliac crest)^39^ and HC was taken at the most protuberant area (i.e. the highest HC value). These anthropometric measurements were performed at the participants’ own home. BMI and waist-to-hip ratio (WHpR) were calculated by dividing the weight (kg) by the square height (m2) and dividing the WC by the HC, respectively.
8. **Hospital Anxiety and Depression Scale (HADS):** The HADS is comprised of two subscales, which are depression and anxiety, and is a valid and reliable self-rating scale that can be used to measure anxiety and depression in both hospital and community settings^40^. The overall score for both the anxiety and depression subscales range from 0 to 21. The Iranian version of the HADS has been found to be acceptable, valid, and reliable^41^.
9. **Duke Social Support Index (DSSI):** The DSSI was developed as a brief and easily administered tool to assess an individual’s level of social support^42^. The DSSI consists of two subscales, social interaction (4 items) and satisfaction with social support (7 items). The reliability and validity of the Persian version has been previously confirmed^43^.
10. **Physical Activity Scale for the Elderly (PASE):** PASE is a brief and easily scored scale designed to assess physical activity among older adults. The PASE score combines information on leisure, household, and occupational activity^44^. The PASE assesses the types of activities typically chosen by older adults (i.e. walking, recreational activities, exercise, housework, yard work, and caring for others) and the Persian version (P-PASE) has been found to be valid and reliable^45^.
11. **The Pittsburgh Sleep Quality Index (PSQI):** The PSQI measures the quality and patterns of sleep in older adults. It contains seven domains, which are subjective sleep quality, sleep latency, sleep duration, habitual sleep efficiency, sleep disturbances, use of sleep medication, and daytime dysfunction over the last month. Each of the mentioned sleep components produces a score ranging from 0 to 3, with 3 indicating the highest level of dysfunction. The total sleep quality score ranges from 0 to 21, with a higher total score indicating worse sleep quality^46^. The psychometric properties of the Persian version of the PSQI have been confirmed^47^.
12. **De Jong Gierveld Loneliness Scale:** This scale is a valid and reliable instrument that is used to assess loneliness in older adults. The scale contains the dimensions of social and emotional loneliness^48^. Scores for each dimension range from 0–3 and the overall loneliness score ranges from 0–6. The Persian version of the Loneliness Scale has been found to be a reliable and valid measure of loneliness in Iranian older adults^49^.
13. **Nutritional habits assessment**: This questionnaire is based on the Traditional Persian medicine (TPM) guidelines regarding nutritional habits (NH). The validity and reliability of the questionnaire has previously been confirmed. The TPM contains 31 questions, with responses being recorded on a Likert scale (“always” to “never”). Scores from the nine subscales (i.e. food combinations, drinking, eating, time of meals, eating and season, eating and bathing, eating and physical activity, and eating and sleeping) are summed to generate a total NH score (maximum score of 150), with higher scores indicating better nutritional habits^50^.
14. **Mizaj (temperament):** Mizaj is the main theory of health and disease in TPM and determines the physical, physiological, and mental characteristics of individuals. It has been demonstrated that mizaj changes with advancing age, so there is a demand for specific alterations which best match their changed needs. For example, it can be made by foods whose nature is opposite to the individual’s present Mizaj. The reliability and validity of the temperament questionnaire has been confirmed by Mojahedi et al.^51^.
15. **Pattern of medication use:** Self-reported data on current medication use was also recorded. The term “medication” refers to conventional drugs, including both prescribed and over-the-counter (OTC) drugs. To investigate the pattern of medication use and the prevalence of polypharmacy (defined as the regular use of five or more drugs per day), participants were asked to show the investigators all products they were currently using.

### Statistical analysis

The proportion of missing data for all of the main outcomes variables was low (about 2%). As such a low percentage of missing data is not likely to affect the study results, the missing data were not imputed^52^. An Independent Samples T-Test was used to compare the means of the normally distributed numeric independent variables. The Chi-square test was used to assess the association between two categorical variables. Descriptive data have been presented as frequency (and percentages) and means (and standard deviations (SDs)) for categorical and continuous variables, respectively. All statistical analyses were conducted using Stata statistical software package (Release 16. College station, TX: StataCorp LP.). A p-value of <0.05 was considered to be statistically significant.

### Ethical concerns

The study protocols were reviewed and approved by the Deputy of the Research Ethics Committee at the Tabriz University of Medical Sciences (Ethical ID: TBZMED.REC.1395.684). Informed consent was obtained from all participants and they were assured about the confidentiality of all information collected. This study was undertaken in accordance with national regulations and ethical guidelines.

## Results

### Baseline characteristics

The socio-demographic characteristics of our sample of 1362 older adults are summarized in Table 1. More than half of the participants were women (56.4%) and in most cases the socio-economic conditions were more favorable among men. Almost half of the studied population were completely illiterate, with the illiteracy rate being significantly higher among women than among men (73.5% vs. 26.5%; *p*<0.001). The majority of the older adults owned their current residence, but women were more often tenants than men (61.7% vs. 38.3%). In terms of marital status, the majority were married, with less than 28% being single. Among those who were single, 87.7% were women. Inequality was also found in terms of living conditions and family type, with women more often in a worse position than men. More than 88% of those living alone were women. The overall prevalence of smoking was less than 10%, but was much higher among men than among women (20.9% vs. 1.5% respectively). Although 27.7% of the participants walked for ≥ 60 min daily, 13.3% did not walk each day. Furthermore, women had a more sedentary lifestyle than men, with no daily walking being reported by 17.1% of women and 8.5% of men. In addition, the proportion of men who walked for at least 60 min each day was also higher than among women (42.4% vs. 16.4%; p<0.001) (Table 1).

**Table 1:** Socio-demographic characteristics of the sample of community-dwelling older adults in Tabriz.

Characteristics		Sex		Total n (%)	*p* value^¥^
		594 Male n (%)	768 Female n (%)		
Age	60–69	305 (51.4)	436 (56.8)	741 (54.4)	0.137
	70–79	201 (33.8)	230 (29.9)	431 (31.6)	
	>=80	88 (14.8)	102 (13.3)	190 (13.9)	
Smoking	Non smoker	379 (63.8)	753 (98.0)	1132 (83.1)	<0.001
	Quit smoking	91 (15.3)	4 (0.5)	95 (6.9)	
	Current smoker	124 (20.9)	11 (1.5)	135 (9.9)	
Education	Illiterate	174 (29.3)	483 (62.9)	657 (48.4)	<0.001
	Primary	186 (31.3)	131 (17.0)	317 (23.3)	
	Secondary	71 (12.0)	47 (6.1)	112 (8.2)	
	Higher education	163 (27.4)	107 (13.9)	270 (19.9)	
Marital Status	Married	548 (92.3)	441 (57.4)	989 (72.6)	<0.001
	Single	46 (7.7)	327 (42.6)	373 (27.3)	
Housing ownership	Tenant	28 (4.8)	45 (5.9)	73 (5.4)	0.377
	Owner	561 (95.2)	717 (94.1)	1278 (94.5)	
Place of birth	Village	279 (47.0)	412 (53.6)	691 (50.7)	0.016
	Town	315 (53.0)	356 (46.4)	671 (49.3)	
Kind of family	Alone	19 (3.2)	145 (18.9)	164 (12.0)	<0.001
	Nuclear	532 (89.7)	516 (67.2)	1048 (77.0)	
	Extended	33 (5.6)	86 (11.2)	119 (8.7)	
	Other	9 (1.5)	21 (2.7)	30 (2.2)	
Health insurance	Covered	571 (96.1)	743 (96.7)	1314 (96.4)	0.320
	Non covered	23 (3.9)	25 (3.3)	48 (3.5)	
Supplementary HI	Covered	320 (53.9)	356 (46.4)	676 (49.6)	0.006
	Non covered	274 (46.1)	412 (53.6)	686 (50.4)	
License & Driving	Current driving	263 (44.9)	24 (3.1)	287 (21.1)	<0.001
	Stopped driving	103 (17.6)	27 (3.5)	130 (9.6)	
	Lack of license	220 (37.5)	717 (93.4)	937 (69.2)	
Head of family	Himself/herself	581 (97.8)	273 (35.6)	854 (62.8)	<0.001
	Spouse	5 (0.8)	435 (56.6)	440 (32.3)	
	Children	8 (1.4)	60 (7.8)	68 (5.0)	
Daily walk	Lack of walking	50 (8.5)	130 (17.1)	180 (13.3)	<0.001
	Up to 15 min	43 (7.4)	132 (17.3)	175 (13.0)	
	15–30 min	125 (21.4)	184 (24.2)	309 (23.0)	
	30–45 min	119 (20.3)	190 (25.0)	309 (23.0)	
	≥60 min	248 (42.4)	125 (16.4)	373 (27.7)	

^¥^*p* Value calculated by Chi-square test.

### Social, health, and disability-related measures

The results of the social measures, health status and disability measures are summarized in Table 2. In terms of sleep quality, women had higher mean PSQI scores than men (5.97 vs. 4.63), indicating worse sleep. Also, the levels of anxiety and depression, as measured by the HADS, were higher among women than men (Depression: 10.63 vs. 9.54; *p*<0.001 and Anxiety: 7.59 vs. 5.79; *p*<0.001). Furthermore, scores from the DSSI, IADL, AMTS, and ADL also revealed worse conditions among women than among men (*p*<0.001 for DSSI, IADL, and AMTS and p=0.031 for ADL). However, there was no substantial difference in the mean SWLS scores between males and females (Male: 16.45 vs. Female: 16.61; *p*=0.466) (Table 2).

**Table 2:** Means for the social measures, health status, and disability measures, by sex.

Variable	Sex		Total	*p* value€
	Male (mean ± SD)	Female (mean ± SD)		
PSQI	4.63±2.70	5.97±2.93	5.39±2.91	<0.001
Depression	9.54±3.20	10.63±3.09	10.15±3.18	<0.001
Anxiety	5.79±4.70	7.59±5.51	6.81±5.24	<0.001
DSSI	23.65±4.50	22.69±4.77	23.11±4.68	<0.001
IADL	7.06±1.81	6.52±2.07	6.76±1.98	<0.001
AMTs	8.96±2.26	6.62±3.17	7.64±3.04	<0.001
ADL	5.73±1.01	5.60±1.24	5.66±1.15	0.031
SWLS	16.61±3.70	16.45±4.07	16.52±3.91	0.466
BMI	27.61±4.33	30.71±5.52	29.34±5.26	<0.001
MNA-SF	12.28±1.99	12.01±1.91	12.13±1.95	0.017

Abbreviations: PSQI: Pittsburgh Sleep Quality Index, DSSI: Duke Social Support Index, IADL: Instrumental Activities of Daily Living, AMTs: Abbreviated mental test score, ADL: Activities of Daily Living, SWLS: Satisfaction with Life Scale, BMI: Body Mass Index, MNA-SF: Mini Nutritional Assessment-Short Form.

€: P-value calculated by Independent Samples T-Test.

### Burden of NCDs

The NCD with the highest prevalence was hypertension (81.0%), followed by cardiovascular diseases (28.2%), and diabetes (26.3%). Males had lower prevalence rates for all measured NCDs, except for stroke, which was more common among older men than among older women (6.9% vs. 5.7%) (Table 3).

**Table 3:** History of major non-communicable diseases amongthe community-dwelling older adults in Tabriz.

Characteristics		Sex		Total n (%)	*p* value^¥^
		594 Male n (%)	768 Female n (%)		
Diabetes	Diabetic	116 (19.5)	243 (31.6)	359 (26.3)	<0.001
	Non diabetic	478 (80.5)	525 (68.4)	1003 (73.7)	
Blood pressure	Hypertensive	436 (73.4)	667 (86.8)	1103 (81.0)	<0.001
	Normotensive	158 (26.6)	101 (13.2)	259 (19.0)	
Stroke	Yes	41 (6.9)	44 (5.7)	85 (6.2)	0.219
	No	553 (93.1)	724 (94.3)	1277 (93.7)	
Cardiovascular	Yes	154 (25.9)	231 (30.1)	385 (28.2)	0.052
	No	440 (74.1)	537 (69.9)	977 (71.7)	

^¥^*p* Value calculated by Chi-square test.

## Discussion

The findings of our population-based survey of older people in Tabriz showed that the prevalence of smoking was less than 10% and that 13.3% reported inadequate physical activity, as measured by daily walking. Almost half of the participants were illiterate and illiteracy was more common among older women than among men. Furthermore, men scored more positively in terms of sleep quality, depression, anxiety, cognitive status, and daily living functions. In contrast, men reported a lower satisfaction with life than women, although this difference was not significant. Moreover, the most common underlying NCD was hypertension.

The prevalence rates for current and ex-smokers were 9.9% and 6.9%, respectively, with smoking being much more common in men than women (20.9% of men vs. 1.5% of women were current smokers). This finding is relatively similar to a cross-sectional survey of 1430 men and 1641 women aged ≥65 from 17 European countries, which reported the prevalence of smoking to be 11.5% and that smoking was more common among men than women (15.3% vs. 8.6%)^53^. Furthermore, a 2014 study among 407 men and 38 women aged 65–79 years old in Portugal found the prevalence rates for daily smoking to be 9.15% (95% confidence interval (CI) 7.30–11.43) among men and 2.43% (95% CI 1.57–3.74) among women^54^. In addition, a nationwide study of smoking patterns among 10,834 Iranians found the proportion of current smokers to be 9.6%, with a higher prevalence being found among men than in women (21.5% vs. 1.1%)^55^. This study also found a higher prevalence rate among those of Turkish ethnicity (12.4%), than that found among other ethnicities. Nevertheless, when interpreting the results of that study it is important to note that their study included participants aged 15–70 years old, unlike the present study which was limited to older adults only^55^.

In terms of education levels, a 2011 cross-sectional survey of 15,069 older adults in Tehran found that 27.0% were illiterate and 14.2% had an academic education^56^. This finding contrasts with our survey, which showed a much higher illiteracy rate in Tabriz (48.4%). This relatively large difference might be due to the larger number of higher educational facilities in Tehran. In accordance with our findings, a 2011 study of 1,350 older residents from five provinces of Iran found significant sex differences in educational level, and the illiteracy rates were reported to be 52.3% in older men and 78.4% in older women^57^. Improving educational levels of the older people should be undertaken, not only because it is an indicator of social development, but also because it can help increase the knowledge of older adults to reduce modifiable risk factors of age-related disorders and Alzheimer’s disease^58^. Considering the inequalities in general education and the high rate of illiteracy among older adults, health promotion and educational activities should be tailored to meet the needs of those who are poorly educated or illiterate. Although general literacy and education are key determinants of health, health literacy, as a distinct form of literacy, is becoming increasingly important for economic and social development^59^. Creating content and providing audio and visual training using the local language and clear communication techniques^60^ are needed to improve health literacy among illiterate or poorly educated people. New information technologies might be able to provide learning opportunities that are more visual and interactive than pamphlets or older health instructions^59^, which can help to increase opportunities to access, understand and use health information^61^.

Several studies have reported significant inequalities in the distribution of education, employment, income, and health services, as well as physical and social activity between men and women^62^. A study of 340 old people in Maku, West Azerbaijan province, showed that 54% were inactive and that physical activity was low (94.2), as measured using the PASE^63^. Furthermore, a study on those aged 60-80 years old from Shiraz showed that an inadequate level of physical activity was significantly higher among women than among men (92.6% vs. 86.6%; p=0.002). Also in accordance with our findings, a study on the physical activity profile of Iranian adults and older adults reported that the highest frequency of physical inactivity was in the >75 age group (73.8%; 95% CI 71.5–76.1%) and this figure was higher in women than among men (87.2% vs. 62.6%)^64^.

In the present study, the mean PSQI score was higher in women than men, meaning that women reported more sleep disturbances than men. A study on the quality of sleep among 400 older people living in the Qazvin province showed that the mean PSQI score was 7.11 and that women had a lower quality of sleep than men (the mean PSQI scores were 7.39 vs. 6.80; p=0.057)^65^. Furthermore, a large-scale population-based study among those living in rural communities in China showed that the prevalence of sleep disturbance was higher in women than in men^66^. A systematic review has shown a relationship between social isolation and poor sleep quality^67^. Therefore, the low quality of sleep found among women in the present study may have been partly due to the high proportion of older women that were living alone.

In terms of psychological factors, a cross-sectional study of 1612 older residents in the north of Iran reported significantly better social support among men than in women, as measured using the DSSI (mean: 10.4 vs. 9.9; *p*=0.001)^68^. Furthermore, a cross-sectional study of 300 older Iranian individuals reported significantly better daily functioning in older men than in older women, as measured by the ADL and IADL (Mean ADL: 18.72 vs. 16.87; *p*<0.001 and IADL: 14.48 vs. 9.60; *p*<0.001)^69^. In contrast, a study of 1067 adults aged 60 and older in Tabriz found an average SWLS level of 14.14, and women were more satisfied than men (*p*=0.01)^70^. The findings of the present study were in accordance with previous comparable research, in that men had higher sleep quality and social support, and only their satisfaction with life was lower than among women.

The present study also found that 26.3% of the older participants had diabetes, which is very similar to the results from a large population-based survey in Tehran, where the prevalence of diabetes was found to be 26.2% among the older adult population. That same study also reported that being female and advancing age were both significantly associated with diabetes^71^. Possible strategies for controlling diabetes include making lifestyle changes, such as encouraging more physical activity, and educating older adults about how to prevent the disease. Likewise, blood sugar levels should be regularly checked in older adults^72^. We reported that approximately 81% of our sample had hypertension, which is considerably higher than previous research in Tabriz, which reported a prevalence of 68% in individuals ≥60 years old^26^. During the period 1980–2012, hypertension was found to be more prevalent in women aged ≥40 years of age, than among men in this age group^73^. In line with previous research, the present study found that hypertension was almost 13% more common in women than among men (86.8 vs. 73.4). The exact reason for this difference is not clearly understood, but it may be due to both biological and social factors^74^. Previous research has reported that decreased estrogen levels, following menopause, results in the dysregulation of nitric oxide synthesis, which is a vasodilator^75^. Furthermore, an increase in arterial stiffness, the expression of angiotensin II receptors, and salt sensitivity may be other underlining factors that induce hypertension in women following menopause^76^. Moreover, the higher prevalence of obesity in postmenopausal women can also predispose them to hypertension^77^. According to findings from the Global Burden of Disease 2019, the prevalence rate of stroke in adults aged over 70 years old was higher in women than among men (8,176.47 (95% uncertainty interval (UI): 6,832.03-9,877.05) vs. 7,438.10 (95% UI: 6,160.54-8,966.32))^78^, while in Tabriz we found stroke to be more common in men than women (6.9% vs. 5.7%).

The strengths of our study include its large sample size and the use of a wide variety of questionnaires to measure several important aspects of health and the social life of the older adults, as well as the use of face-to-face interviews. However, our study also had several limitations. One of these limitations relates to the cross-sectional nature of the study, which limits our understanding of the relationships between variables. Also, our sample only included residents of Tabriz city and excluded the rural areas. Furthermore, we only evaluated four kinds of NCDs (i.e. cardiovascular disease, stroke, hypertension, and diabetes), omitting other NCDs that cause a high burden in Iran, such as neoplasms, respiratory diseases (e.g. chronic obstructive pulmonary disease), mental disorders, musculoskeletal disorders, and other neurological disorders (e.g. Alzheimer’s disease, Parkinson’s disease, and multiple sclerosis)^79^. In addition, we used self-reported data to measure underlying diseases and the use of medications, which might not be completely reliable. Finally, it is also important to note that our findings are not likely to be representative of the entire Iranian population, since our sample was from a limited cultural, geographical, and ethnic group of older Iranian adults.

## Conclusion

The findings of the present research have shown that there are significant sex differences among older adults in Iran. Paying attention to health and social inequalities among older men and women is important for several reasons, including the: importance of addressing inequality, vulnerability of older adults, growing number and proportion of older adults, and the increasing feminization of the aged globally. A better understanding of these disparities will help community leaders and policymakers with social planning and to address some of the health challenges facing older people. This information can be used to target programs and to advocate for system improvements that promote health equity. Addressing sex differences in health and health care access requires new attitudes at all levels of the health system.

## Data Availability

Data gathered for the study is available from the corresponding author.
